# Flexible endoscopic evaluation of swallowing (FEES) for neurogenic dysphagia: training curriculum of the German Society of Neurology and the German stroke society

**DOI:** 10.1186/s12909-016-0587-3

**Published:** 2016-02-25

**Authors:** Rainer Dziewas, Jörg Glahn, Christine Helfer, Guntram Ickenstein, Jochen Keller, Christian Ledl, Beate Lindner-Pfleghar, Darius G. Nabavi, Mario Prosiegel, Axel Riecker, Sriramya Lapa, Sönke Stanschus, Tobias Warnecke, Otto Busse

**Affiliations:** Department of Neurology, University Hospital Münster, Albert-Schweitzer-Campus 1, 48149 Münster, Germany; Department of Neurology, Johannes Wesling Klinikum Minden, Minden, Germany; ENT and Neurology Departments, Vivantes Klinikum Neukölln, Berlin, Germany; Department of Neurology and Stroke Unit, HELIOS Klinikum Aue, Aue, Germany; St. Martinus Hospital, Clinic for Acute Geriatrics, Düsseldorf, Germany; Schön Klinik Bad Aibling, Bad Aibling, Germany; Department of Neurology, University of Ulm, Ulm, Germany; Department of Neurology, Vivantes Klinikum Neukölln, Berlin, Germany; German Society for Dysphagia, Munich, Germany; Reha Nova, Clinic for neurological and neurosurgical rehabilitation, Cologne, Germany; Department of Neurology, ZNN, Johann Wolfgang Goethe University, Frankfurt am Main, Germany; Hospital zum Heiligen Geist, Kempen, Germany; DSG and DGN Office, Reinhardtstraße 27 C, 10117 Berlin, Germany

**Keywords:** Nervous system diseases, Flexible Endoscopic Evaluation of Swallowing, Muscular diseases, Stroke, Clinical competence

## Abstract

**Background:**

Neurogenic dysphagia is one of the most frequent and prognostically relevant neurological deficits in a variety of disorders, such as stroke, parkinsonism and advanced neuromuscular diseases. Flexible endoscopic evaluation of swallowing (FEES) is now probably the most frequently used tool for objective dysphagia assessment in Germany. It allows evaluation of the efficacy and safety of swallowing, determination of appropriate feeding strategies and assessment of the efficacy of different swallowing manoeuvres. The literature furthermore indicates that FEES is a safe and well-tolerated procedure. In spite of the huge demand for qualified dysphagia diagnostics in neurology, a systematic FEES education has not yet been established.

**Results:**

The structured training curriculum presented in this article aims to close this gap and intends to enforce a robust and qualified FEES service. As management of neurogenic dysphagia is not confined to neurologists, this educational programme is applicable to other clinicians and speech–language therapists with expertise in dysphagia as well.

**Conclusion:**

The systematic education in carrying out FEES across a variety of different professions proposed by this curriculum will help to spread this instrumental approach and to improve dysphagia management.

## Background

Neurogenic dysphagia is one of the most frequent and life-threatening symptoms of neurological disorders. Swallowing impairment is observed in at least 50 % of all patients with ischaemic or haemorrhagic stroke [1–3]. These patients have a three-fold increased risk of developing early aspiration pneumonia, and their mortality is significantly higher than that of non-dysphagic stroke patients [2]. Similar data have been published for severe traumatic brain injury, in which the incidence of clinically relevant dysphagia is approximately 60 % [4]. In this patient population, the occurrence of dysphagia is associated with significantly longer artificial respiration and prolonged artificial nutrition [5]. In patients with Parkinson’s disease, neurogenic dysphagia is also a major risk factor for the development of pneumonia, the most frequent cause of death in this patient group [6]. In addition, swallowing disorders in these patients typically lead to major and long-term reduction in quality of life, insufficient medication intake and pronounced malnutrition [7]. Around 20–30 % of all patients with dementia have severe dysphagia with silent aspiration, which goes unnoticed by the patient themself [8–10]. Up to 30 % of all amyotrophic lateral sclerosis (ALS) patients present with swallowing impairment at diagnosis [11] and practically all of them develop dysphagia as the disease progresses. In 15 % of all cases, myasthenia gravis manifests itself with swallowing impairment. As the illness progresses, over 50 % of all patients are affected, and in more than 50 % of cases, a myasthenic crisis is preceded by dysphagia [12]. Patients with inflammatory muscle disorders are also often subject to swallowing impairment. The frequency is approximately 20 % in dermatomyositis, 30–60 % in polymyositis, and between 65 and 86 % in inclusion body myositis [13]. Finally, dysphagia also represents an important diagnostic and therapeutic challenge in the intensive care unit. Regardless of the primary illness, 70–80 % of patients requiring prolonged mechanical ventilation present, at least temporarily, with significant swallowing impairment and aspiration after succesful weaning, probably due to a critical illness polyneuropathy and structural changes caused by the artificial airway like edema of the arytenoids [14, 15]. This impairment not only necessitates prolonged artificial nutrition, but is also linked to serious complications, such as pneumonia and the necessity for reintubation. In addition, it is an independent predictor of increased mortality [16].

### Flexible endoscopic evaluation of swallowing (FEES)

The above-mentioned data indicate that swallowing impairment is a nearly ubiquitous problem in neurology. Affected patients are either treated on an outpatient basis, e.g. in specialised consultations for movement or neuromuscular disorders, or on an inpatient basis, where dysphagia is observed at all levels of care, from the general ward to the intermediate care/stroke unit and the intensive care unit.

Traditionally, the first step in systematic evaluation of dysphagia is a clinical swallowing examination performed by appropriately qualified speech and language therapists (SLT). However, the validity and the reliability of this clinical approach are generally insufficient. Particularly the pharyngeal phase of swallowing and silent aspiration, often seen in patients with neurogenic dysphagia, are difficult to detect using this approach [17]. Therefore, leading experts in this field often express reservations regarding the value of the clinical swallowing examination, and consider an additional instrumental dysphagia assessment to be an absolute necessity [18–20].

At present, the flexible endoscopic evaluation of swallowing (FEES) is probably the most commonly chosen method for the objective assessment of swallowing in Germany. It is used in more than 50 % of all certified stroke units [21] as well as in numerous acute and rehabilitation clinics. Over the past years, the significance of FEES has increased. This is also reflected by the fact that in 2010, the German Institute of Medical Documentation and Information (DIMDI) defined a separate code for this examination in Chapter 1 ‘Diagnostic Measures’ of the official classification for operations and procedures (1-613: flexible endoscopic evaluation of swallowing).

FEES was first described in 1988 by the American SLT Susan Langmore and colleagues [22] and defined as a procedure separate from conventional otorhinolaryngoscopy, which lacks evaluation of swallowing. In Anglo-American countries, FEES is therefore, to this day, predominantly performed by SLTs [23, 24].

Originally, FEES was conceived as an alternative to the historical gold standard, the X-ray-based videofluoroscopic swallowing study (VFSS), to be used when VFSS was either not available or not applicable. However, besides being increasingly used in a clinical context, FEES has, within the last 15 years, established itself as an independent and efficient method alongside VFSS [25, 26]. In the meantime, numerous studies have shown that FEES is at least as efficient as VFSS, in some studies even superior to VFSS, in terms of detecting critical events, such as penetrations, aspirations and residues [27–29]. Additionally, FEES is highly reliable, a fact underlined by an inter-rater reliability of more than 90 % in various studies [30, 31]. In terms of day-to-day practicality, the advantages of FEES are that i) it can be performed at the bedside, thus facilitating examination of severely motor-impaired, bedridden or uncooperative patients; ii) follow-up examinations can be performed at short notice and, if necessary, frequently; and iii) oropharyngeal secretion management and efficacy of cleaning mechanisms, such as coughing and throat clearing, can be assessed simply and directly [32]. Today, FEES and VFSS are therefore considered to be complementary methods.

Visualisation of the swallow by means of FEES involves introducing a flexible nasopharyngolaryngoscope transnasally into the pharynx via the inferior or middle nasal meatus. FEES provides an extensive picture of the pharyngeal phase of swallowing and enables the detection of indirect signs of impairment within the oral and oesophageal phases. The aims of FEES are, in particular, to identify pathological movement patterns, to assess the effectiveness and safety of swallowing, to determine suitable food consistencies or forms of nutrition and to guide the use of therapeutic manoeuvres for each patient. Data show that FEES is an exceedingly well-tolerated and safe examination. Of 6000 examinations performed, only 222 (3.7 %) were terminated prematurely at the patient’s request [25]. The most commonly cited side effect is self-limiting nosebleed, observed in approximately 1 % of all cases in a mixed patient population [33–35]. Severe side effects, such as fulminant aspiration associated with respiratory insufficiency or vasovagal reaction, were not described in these studies. Laryngospasm occurred in less than 1 % of all examinations. These results were reproducible in a group of acute stroke patients. The self-limiting nosebleed rate (6 %) was higher than in other studies, but no serious side effects were observed. Measured autonomic reactions (heart frequency and blood pressure fluctuations) were mild [36]. In terms of patient safety and invasiveness, FEES is certainly less hazardous and stressful than the insertion of a nasogastric tube [37] and nasotracheal suctioning, in particular.

FEES has been successfully applied in diverse patient populations and disease patterns. Among others, studies describing stroke and traumatic brain injury patients [30, 38], patients with neurodegenerative (dementia, Parkinson’s disease) [10, 39] and neuromuscular diseases (ALS, Kennedy’s disease, inclusion body myositis) [40, 41] as well as head and neck cancers [42] have been published. FEES is also being increasingly applied in paediatrics, geriatrics and intensive-care medicine [43, 44].

### The training curriculum

Despite the numerous possible applications of FEES in neurology and the undisputed strong need for qualified dysphagia assessment in this area of expertise, in Germany this technique is not yet systematically taught in neurological residency programmes or training programmes for SLTs. The FEES curriculum described hereafter aims to close this gap. In particular, two main objectives are linked to this step: on the one hand, the definition of quality standards aims to guarantee consistent and high standard performance of FEES. In the long run, the intended standardisation of terminology, examination algorithms and interpretation of results will not only facilitate professional communication within a given hospital, but will also contribute to the optimisation of understanding between the various sites involved in the treatment of an individual patient over time, e.g. acute clinic, rehabilitation clinic, outpatient care. On the other hand, the introduction of a formal curriculum also leads to a valorisation of FEES. Learning this method will thus become more attractive and will turn into an independent, clinically relevant and sought-after qualification.

While this curriculum is therefore of interest to all clinicians practicing in Germany, it could also be used by other countries after adapting it to their specific needs.

The diagnostics and therapy of swallowing disorders are relevant to many disciplines. This training curriculum is therefore not only intended for neurologists but is open to all clinicians with an interest in this topic. It also offers SLTs, in particular, the opportunity to acquire qualifications in the area of instrumental dysphagia assessment and to expand their range of activities.

The curriculum and the qualification levels were developed in keeping with the guidelines of the Royal College of Speech and Language Therapists (RCSLT) [23] and the guidelines of the American Speech–Language–Hearing Association (ASHA) [24].

At this point, attention should explicitly be drawn to the fact that the present curriculum addresses neurogenic dysphagia. Therefore, neither the diagnostics of structural changes in the mouth and throat (e.g. tumours or anatomical variants), nor the examination of swallowing disorders due to such ailments (e.g. structural changes after surgery or irradiation) are dealt with in this training programme.

### Prerequisites

The following prerequisites have been defined for qualification in the area of FEES within this curriculum:

- One year of clinical practice focused on the care of neurological patients is required for physicians and two years of that same clinical experience for SLTs. Three months of this period shall be completed in a neurological department.
- Along with the acquisition of the FEES certificate, the following requirements, specific to each professional group, must be fulfilled in order to attain the status of a FEES instructor: SLTs must be in possession of at least 5 years of experience in the area of diagnostics and therapy of neurogenic dysphagia. Clinicians must have acquired a specialist title.
- Willingness to participate in a trans-regional FEES registry.

### Qualification levels

Training in the field of FEES is divided into two stages: the FEES certificate and the FEES instructor certificate.

### FEES certificate

**Fig. 1:**
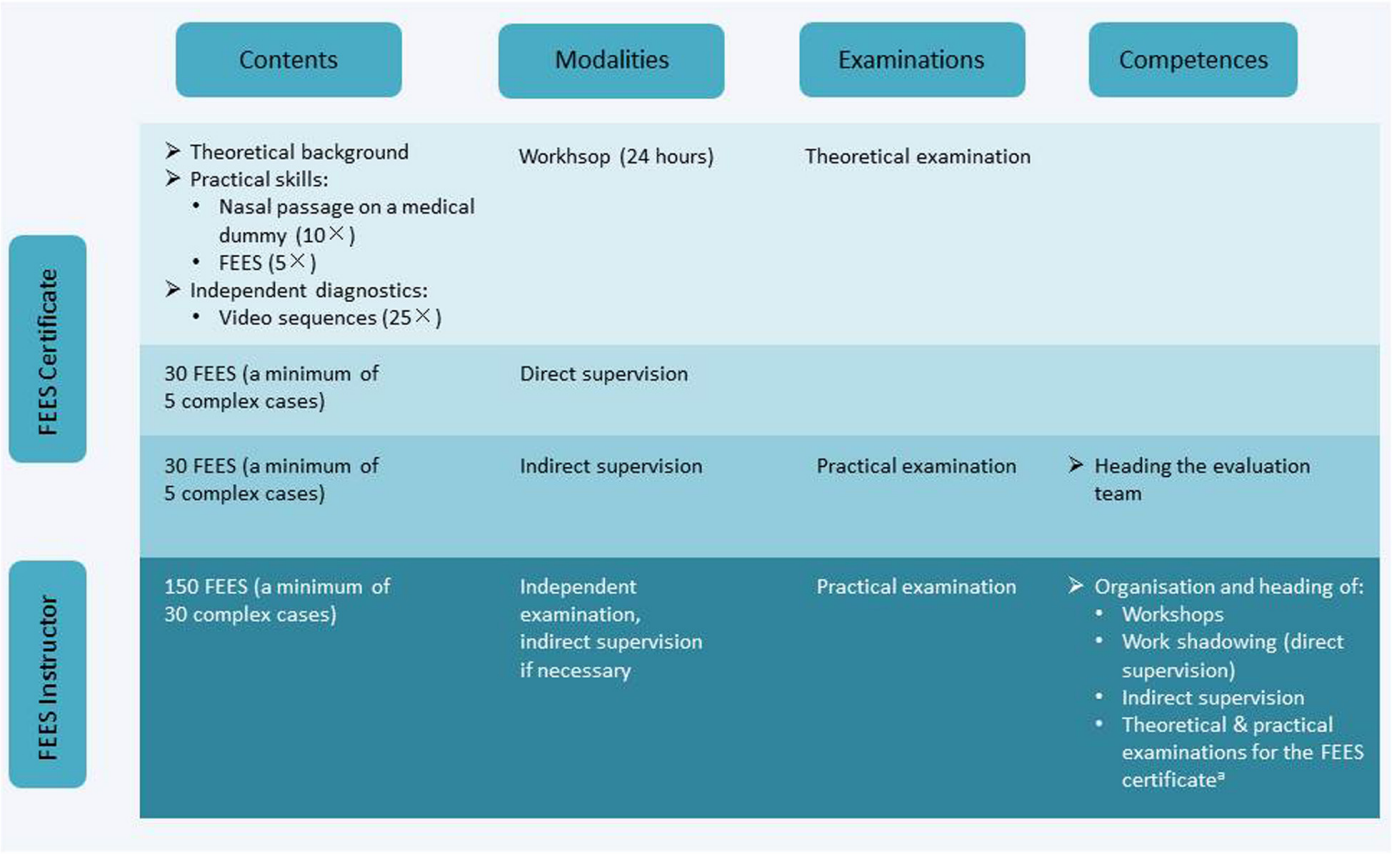
Detailed overview of educational steps leading to the FEES certificate and FEES instructor certificate. ^a^Applying for an authorisation to administer FEES-instructor examinations implies a minimum of 2 years of activity in this field, the verifiable performance of at least 500 FEES, the participation in the organization, and realisation of, at least one curricular FEES workshop, the training of at least five FEES-certificate holders and, optionally, a relevant scientific occupation

The holder of a FEES certificate is entitled to perform FEES, to prepare the related report and to define clinical consequences in collaboration with the treatment team. Training consists of the following sections (please also refer to Fig. 1):

- Workshop
- FEES under direct supervision
- FEES under indirect supervision

### Workshop

At least 24 hours of advanced training will serve to impart theoretical and practical knowledge. The obligatory topics of the theoretical course are listed in Table 1. Additionally, handling of the endoscope will be practiced (a minimum of 10 times) using a medical dummy. The participants will then improve their technical skills by means of reciprocal examinations (a minimum of five times). Interpretation of typical endoscopic findings will be practiced using suitable video sequences. Participants will analyse at least 25 sequences independently and discuss their findings with the tutors. Training will end with a theoretical test comprising 25 multiple-choice questions pertaining to the contents of the curriculum. To pass the test, 60 % of the questions must have been answered correctly. Participants who fail the test can request an oral examination.

**Table 1:** Contents of the basic workshop

A) Basics
• History of FEES
• Aims of the evaluation
• Indications
• Contraindications
• Limits
• Examination procedure
• Distribution of tasks and responsibilities within the examination team
• Alternative instrumental dysphagia assessments and their indications
○ Videofluoroscopic swallow study
○ Pharyngeal and oesophageal manometry
B) Diseases
• Neurovascular diseases (e.g. ischaemic stroke)
• Neurodegenerative diseases (e.g. Parkinson’s disease, dementia)
• Neuromuscular diseases (e.g. ALS, polymyositis)
• Neurotraumatology (e.g. traumatic brain injury)
• Neuro-oncological diseases (e.g. gliomas, paraneoplastic diseases)
• Neuroinfectious diseases (e.g. brainstem encephalitis)
• Age-related changes in the swallowing mechanism (presbyphagia)
• Differential diagnosis of neurogenic dysphagia (e.g. cervical spine surgery, Morbus Forestier, disobliteration of the internal carotid artery, laryngeal reflux, Zenker’s diverticulum)
C) Equipment
• Flexible endoscope
○ Fibre endoscope
○ Video endoscope
• Light source
• Video camera
• Processing software
• Consumables
• Hygiene and cleansing
D) Preparations
• Patient information
• Patient positioning
• Local anaesthesia
• Nasal decongestant
• Defogging
• Emergency management
E) Endoscope handling and placement
• Holding and operating the endoscope
• Nasal passage
• Velum
• Oropharynx/hypopharynx and larynx
○ Home position
○ Close view
F) Standard FEES protocol
• Anatomic observation
○ Stenosis of the nasal meatus
○ Velopharyngeal incompetence
○ Pharyngeal stenosis (post radiation)
○ Post-operative findings
○ Mucosal abnormalities
○ Oedema
○ Signs of gastro-oesophageal reflux
○ Irregular position of gastric tube
○ Saliva pooling
○ Abnormal position of epiglottis, arytenoid cartilage and glottis
• Physiological examination
○ Velopharyngeal closure
○ Movement of the base of the tongue
○ Epiglottis inversion
○ Pharyngeal wall contraction
○ Vocal cord and vestibular fold movement
○ Sensory functions
• Evaluation of swallowing
○ Choice of consistency depending on the problem at hand
○ ‘White-out’ characterisation and post-swallow stage
○ Identification of the salient findings
– Oral bolus control, leaking
– Delayed swallowing reflex
– Residues
– Penetration
– Aspiration
– Temporal characteristics of penetration and aspiration (predeglutitive, intradeglutitive or postdeglutitive)
– Adequacy of clearance effort
○ Identification of the main pathomechanisms
• Evaluation of different therapeutic manoeuvres
• Evaluation and interpretation of the examination
○ Classification
○ Degrees of severity
○ Therapeutic consequences (e.g. nutrition management, rehabilitation)
• Indications for referral to further medical departments (e.g. otolaryngology, enterology, phoniatrics)
G) Neurological examination protocols
• FEES protocol for stroke patients
• FEES tensilon test
• Fatigable swallowing test
• FEES L-dopa test
• Decannulation protocol

### FEES under direct supervision

The second phase involves performing FEES under direct supervision. Handling of the endoscope as well as planning of the interventions will be practiced during 30 examinations, and concise reports of the findings will be prepared in each individual case. These will include standard cases as well as a minimum of five complex cases. The latter will include patients with compromised respiratory function, tracheotomised patients, patients whose ability to cooperate is impaired due to ailments such as aphasia or an acute confusional state, as well as patients displaying motor restlessness, caused by, for example, a movement disorder (see Table 2).

**Table 2:** Characteristics of complex patients

Respiratory impairment	
Tracheostomy	
Restlessness (Parkinson’s disease, dystonia, delirium)	
Limited understanding of the situation (severe aphasia due to stroke or encephalitits)	
Fluctuating vigilance	

### FEES under indirect supervision

During the last stage of the education, 30 endoscopic examinations of swallowing will be performed independently and documented in the training record book. Five will involve complex cases. The instructor will be available for questions and will also discuss critical findings with the trainee.

**Fig. 2:**
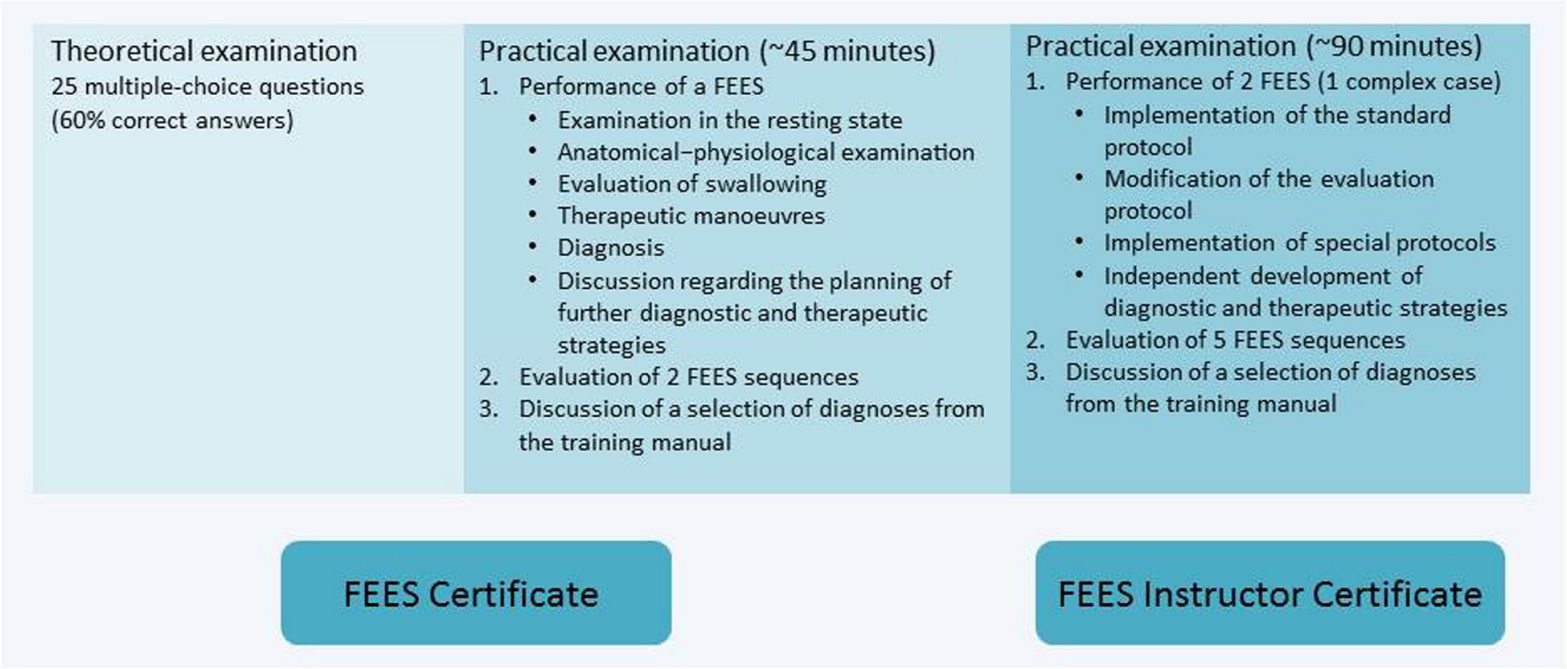
Examination components of the FEES certificate and FEES instructor certificate

The education ends with a practical examination, which involves performing FEES independently. In addition, a report should be generated and further diagnostics, where necessary, as well as the appropriate therapy should be planned by the examinee. The test also comprises assessment and diagnosis of three additional FEES sequences prepared by the examiner. Finally selected findings recorded during the previous training period are discussed with the examinee (see Fig. 2).

### FEES instructor certificate

The FEES instructor possesses extensive knowledge, skills and authorisations. He heads the examination team and has the ability to independently assess all cases, including complex ones. He is licensed to organise FEES-training seminars, can offer work-shadowing opportunities and is entitled to administer the theoretical and practical FEES-certificate examinations.

After the FEES-instructor qualification has been attained, it is possible, through continuing education, to become an authorised examiner of future FEES instructors (see below).

For becoming a FEES instructor further systematic practical training is required that comprises of a minimum of 150 FEES, 30 of which must pertain to complex cases. These evaluations, including complications, must be documented in the FEES training record book. Difficult diagnoses are to be discussed with the responsible instructor.

At the end of this educational period, a practical examination will be taken in an external hospital. This test includes two FEES, one of which must be a complex case. Besides implementing the standard FEES protocol, the examinee must also adapt the examination as needed without external help, explain his examination procedure and be able to implement special FEES protocols. The examinee must also be able to develop diagnostic and therapeutic strategies without assistance. Additionally, he must assess five video sequences prepared by the examiner. Furthermore, findings documented throughout the preceding training period are discussed during the examination (see Fig. 2). Apart from that the candidate must also be able to explain and substantiate the FEES routine established in his institution using appropriate documents (such as diagnosis forms, clinical algorithms).

**Fig. 3:**
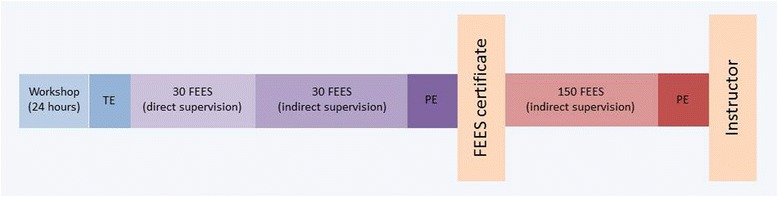
Brief overview of the FEES certificate and FEES instructor certificate. TE, theoretical examination; PE, practical examination

A person who has successfully passed the FEES-instructor examination and has actively worked at least 2 years in this function can, in turn, apply for the authorisation to administer FEES-instructor examinations. Prerequisites include having performed at least 500 FEES, having participated in the organisation and realisation of at least one curricular FEES seminar, the training of at least five FEES-certificate holders and, optionally, a relevant scientific occupation. The complete FEES-training curriculum is summarised in Fig. 3.

Regardless of the level of training, the required endoscopies can be performed in the candidate’s own institution and/or within the scope of work-shadowing opportunities and workshops in external institutions. Work shadowing is especially meaningful during the initial stage of training, during which the mediation of technical skills, requiring intensive personal supervision, is particularly important. For advanced users, workshops offering discussions on complex cases could be an option, as these are an ideal setting in which to discuss rare, subtle or difficult-to-interpret findings in a focussed manner.

### Training record book

Complete documentation of the FEES education in the training record book is required.

### Task assignment and delegation

As stated above, this curriculum is also open to SLTs, a group of non-medical professionals. For this reason, the aspects of task assignment and delegation are briefly addressed here. In principle, this curriculum encourages the performance of FEES by a team of physicians and SLTs. Tasks can be assigned flexibly, taking into account the training level of each person involved. In all cases, however, a physician who is familiar with the disease at hand should be included in the interpretation of the findings and the planning of diagnostic and therapeutic consequences. In the authors’ and the involved professional associations’ view, the practical performance of the endoscopy can be delegated to a qualified SLT by the physician. The basic principles regarding the delegation of medical tasks to non-medical personnel must be respected. This above all implies that the responsible physician must be at calling distance and be able to intervene immediately in case of an emergency.

### Applying for the FEES certificate and the FEES-instructor status

Following completion of the different educational steps of this curriculum, requests for the FEES certificate and the FEES-instructor status can be submitted to the German Society of Neurology (DGN). The applications are evaluated by the “FEES curriculum” task force of the DGN and DSG.

### Accreditation of curricular FEES training courses

FEES certificate training events planned by FEES instructors must be evaluated and accredited by the “FEES curriculum”task force of the DGN and DSG.

### Transitional arrangement

Until 31 December 2015, the FEES certificate and the FEES-instructor status, including full entitlement to administer examinations leading to the FEES instructor status, can be granted within the framework of a transitional arrangement under the conditions listed below.

FEES certificate:

- Proof of training in an institution with FEES expertise
- 2 years of experience in the area of FEES with patients presenting neurogenic dysphagia
- A minimum of 200 performed evaluations

FEES instructor:

- 5 years of experience in the area of FEES with patients presenting neurogenic dysphagia
- A minimum of 500 performed evaluations
- Establishment of examination standards within the applicant’s hospital
- Internal advanced training for staff members
- For physicians: specialist title

## Conclusion

Neurogenic dysphagia is one of the most frequent and prognostically relevant neurological deficits in a variety of disorders, such as stroke, parkinsonism and advanced neuromuscular diseases. Flexible endoscopic evaluation of swallowing (FEES) is now probably the most frequently used tool for objective dysphagia assessment in Germany. The systematic education in carrying out FEES across a variety of different professions proposed by this curriculum will help to spread this instrumental approach and to improve dysphagia management.

## Abbreviations

ASHA: American speech–language–hearing association
DGN: German society of neurology (Deutsche Gesellschaft für Neurologie)
DSG: German stroke society (Deutsche Schlaganfallgesellschaft)
FEES: Flexible endoscopic evaluation of swallowing
RCSLT: Royal College of speech and language therapists
SLT: Speech language therapist
VFSS: videofluoroscopic swallowing study
